# The Effects of Simvastatin or Interferon-α on Infectivity of Human Norovirus Using a Gnotobiotic Pig Model for the Study of Antivirals

**DOI:** 10.1371/journal.pone.0041619

**Published:** 2012-07-23

**Authors:** Kwonil Jung, Qiuhong Wang, Yunjeong Kim, Kelly Scheuer, Zhenwen Zhang, Quan Shen, Kyeong-Ok Chang, Linda J. Saif

**Affiliations:** 1 Food Animal Health Research Program, Ohio Agricultural Research and Development Center, Department of Veterinary Preventive Medicine, The Ohio State University, Wooster, Ohio, United States of America; 2 Diagnostic Medicine and Pathobiology, College of Veterinary Medicine, Kansas State University, Manhattan, Kansas, United States of America; The University of Hong Kong, China

## Abstract

The lack of an animal model for human norovirus (HuNoV) has hindered the development of therapeutic strategies. This study demonstrated that a commonly used cholesterol-lowering statin medication, simvastatin, which increases HuNoV replication in an *in vitro* replicon system, also enhances HuNoV infectivity in the gnotobiotic (Gn) pig model. In contrast, oral treatment with interferon (IFN)-α reduces HuNoV infectivity. Young piglets, all with A or H1 histo-blood group antigens on enterocytes, were treated orally with 8 mg/kg/day of simvastatin; 5 days later, the pigs were inoculated orally with a GII.4 HuNoV (HS194/2009/US strain) and then treated with simvastatin for 5 more days. Simvastatin induced significantly earlier onset and longer duration of HuNoV fecal shedding in treated pigs, frequently with higher fecal viral titers. Simvastatin impaired poly (I:C)-induced IFN-α expression in macrophages or dendritic cells, possibly due to lowered toll-like receptor (TLR) 3 expression; however, the mechanisms were not related to interferon regulatory factor 3 or nuclear factor kappa B signaling pathway. Thus, the enhanced, earlier infectivity of HuNoV in simvastatin-treated pigs coincided with the inhibitory effect of simvastatin on innate immunity. In contrast to the increased HuNoV shedding that simvastatin induced, viral shedding during the treatment period was reduced or curtailed in the HuNoV-inoculated pigs pre-treated/treated with human IFN-α. Our findings are the first to indicate that IFN-α has potential as antiviral therapy against HuNoV. Based on these intriguing and novel findings using the Gn pig model, we confirmed that HuNoV infectivity is altered by treatment with simvastatin or IFN-α. Collectively, these findings indicate that Gn pigs are a useful model to test immunomodulators or efficacy of antivirals against HuNoV.

## Introduction

Human norovirus (HuNoV), a single-stranded, positive sense RNA virus, is a member of the *Caliciviridae* family. This virus is the leading pathogen causing food- or water-borne gastroenteritis [1]. HuNoVs are estimated to cause 54 million cases of illness annually in the US, and they account for approximately 58% of the foodborne illnesses caused by 31 different bacteria, parasites and viruses [2]. Clinical and pathological features of HuNoV infections include: i) a short incubation period (10–51 hr) prior to onset of clinical signs such as vomiting and diarrhea, although asymptomatic infections occur frequently [3]; ii) acute and self-limiting infection, but often with prolonged fecal virus shedding [1]; and iii) lymphocytic, atrophic enteritis [4].

HuNoVs are classified into 3 distinct genogroups (GI, GII, and GIV), which are further subdivided into 26 or more different HuNoV genotypes [5], [6], [7]. In the last 15 years, the GII.4 HuNoVs have been responsible for the majority of HuNoV outbreaks, possibly due to several viral and host factors that have been reviewed recently [8]: i) the broader binding of host receptor to GII.4 HuNoVs, ii) incomplete herd immunity against GII.4 or its variants, and iii) higher mutation rate of their polymerases. In the viral capsid protein, the P2 domain is the most protruding and variable region. It is believed to recognize host cellular receptors and to contribute to establishment of viral infection. Histo-blood group antigens (HBGAs) are considered as cellular receptors or co-receptors that determine host susceptibility to certain HuNoVs. Individuals of blood type A or O (H) of secretors were more susceptible to GI.1/Norwalk/1968/US virus infection than individuals who were non-secretors or secretors of blood type B [9], [10]. However, an emerging HuNoV, GII.12 strain did not bind to HBGAs when tested *in vitro* [11]. These observations imply a host factor affecting infection by certain genogroups or genotypes of HuNoV.

HuNoV infection is generally self-limiting, but it can induce severe illness and fatal disease in immunocompromised patients, specifically, organ recipients receiving long-term chemotherapy or hematopoietic stem cell transplantation [12], [13]. The young and elderly are also at risk, due to their high exposure rates to HuNoV infection in community settings (child care centers, nursing homes, hospitals, etc). Notably, the common use of statin medications, which lowers serum cholesterol levels and prevents cardiovascular disease, is a significant risk factor for exacerbating HuNoV disease severity and increasing the related fatality rates [14]. These conditions require effective therapeutic strategies against HuNoV infection. However, the lack of small animal model for HuNoVs has hindered development and testing of HuNoV antivirals or vaccines [15].

Gnotobiotic (Gn) pigs are susceptible to oral infection by a GII.4 HuNoV strain (HS66/2001/US) [16], [17] and an emerging GII.12 HuNoV strain (HS206/2010/US) [11]. Most HuNoV-infected Gn pigs shed virus in feces. The incubation period (12–48 hrs) for GII.4 HuNoV in Gn pigs was similar to that (19–41 hrs) observed in humans experimentally infected with the GII.2 Snow Mountain virus [16], [18]. The longer fecal HuNoV shedding in Gn pigs infected with the GII.12 HS206 strain was similar to that observed in human cases [11]. Also, like humans possessing secretor phenotype, Gn pigs express A or H1 HBGA on enterocytes. Different HBGA phenotypes (A or H) were shown to influence susceptibility of Gn pigs to HuNoV infection [19].

In a Norwalk virus (GI.1) replicon-harboring cell system, the viral RNA and protein levels were increased after treatment with cholesterol lowering drugs, such as simvastatin, that act as 3-hydroxy-3-methylglutaryl-coenzyme A (HMG**-**CoA) reductase inhibitors [20]. After statin treatment, reduced cellular cholesterol levels are followed by high expression of low-density lipoprotein receptor (LDLR) gene that compensates for the lower cellular cholesterol levels. These results indicated that cholesterol pathways may be associated with enhanced replication of HuNoV *in vitro*, although the related mechanisms are unclear. Statins are also involved in a variety of immune responses and are immunosuppressive. They inhibit major histocompatability complex (MHC) class II expression on antigen presenting cells in mice, promote the generation of Foxp3+ T regulatory cells in mice, and impair lipopolysaccharide-induced toll-like receptor (TLR) 4-mediated inflammatory responses in human embryonic kidney-293 cells (HEK-293) [21], [22], [23].

The aim of our study was to determine if statins also enhance HuNoV infectivity *in vivo* in our Gn pig model of HuNoV infection [16]. The Gn pigs were treated with high-doses of simvastatin and then inoculated with GII.4 HuNoV (HS194/2009/US strain). We further investigated if the enhanced, earlier infectivity of HuNoV seen in simvastatin-treated pigs correlated with inhibitory effects of simvastatin on innate immunity. Finally, we investigated the effect of an innate immunity mediator, IFN-α on HuNoV infectivity in the Gn pig model.

## Results

### Simvastatin Treatment Lowered the Serum Cholesterol Level in Gn Pigs and Resulted in Increased Early LDLR Gene Expression in a Porcine Enterocyte Cell Line (IPEC-J2)

**Figure 1:**
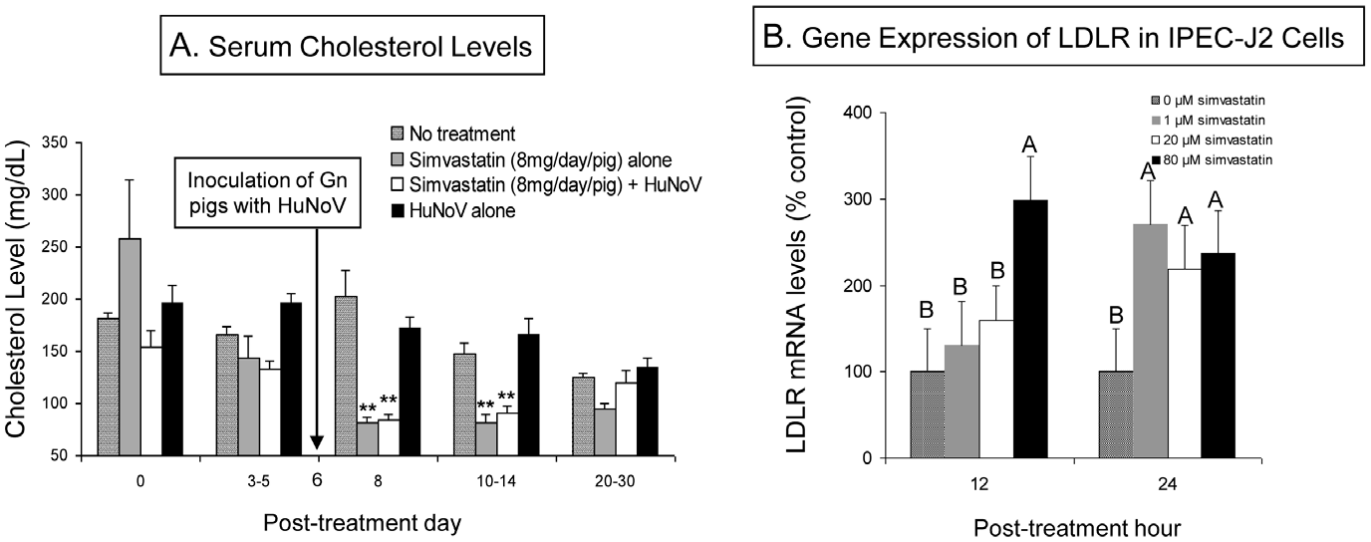
Simvastatin treatment lowers the serum cholesterol level in Gn pigs and results in increased early LDLR gene expression in IPEC-J2 cells. (**A**) Total serum cholesterol levels were assessed in all animals of 3 independent trials (*n* = 6 to 11 pigs at each time-point for Simvastatin + HuNoV and HuNoV alone; n = 2 to 4 pigs at each time-point for Mock and Simvastatin alone) by using an Amplex Red cholesterol assay kit. Pigs were inoculated with HuNoV at 6 days after simvastatin treatment began, i.e. when lower serum cholesterol levels were detected in treated animals compared to pre-treatment levels. Each bar represents the mean ± SEM. **P<0.01 for simvastatin treatment groups vs Mock (or HuNoV alone) at each time-point. (**B**) LDLR gene expression levels in IPEC-J2 cells treated with various concentrations (0, 1 µM, 20 µM, and 80 µM) of simvastatin. LDLR gene expression was analyzed by qRT-PCR at 12 and 24 hours after treatment. Each bar represents the mean ± SEM. Different letters denote significant differences among groups at each time-point (ANOVA test, *P*<0.05). Data from 2 independent experiments were combined, and each test was performed in triplicate.

Serum cholesterol levels were monitored to measure the pharmacological activity of simvastatin in Gn pigs. Simvastatin-treated pigs had significantly decreased serum cholesterol levels (81.5±7.5 to 90.4±7.4 mg/dL) at 8 to 14 days after treatment began, which were 1.6 to 2.4 times lower than the levels in the untreated pigs (147.0±11.0 to 202.3±25.5 mg/dL) during the same period (Fig. 1A).

We further investigated if a reverse relationship existed between the cholesterol level and the LDLR gene expression in a porcine jejunal epithelial cell line, IPEC-J2, because intestinal epithelial cells are a cell type critical for initiation of HuNoV infection [16]. Cells were treated with multiple concentrations (0 µM, 1 µM, 20 µM, and 80 µM) of simvastatin, and LDLR gene expression was analyzed by quantitative real-time RT-PCR (qRT-PCR). At 12 hours after treatment with 80 µM simvastatin, LDLR gene expression levels in IPEC-J2 cells were significantly increased compared to 0 to 20 µM treated groups (Fig. 1B). At 12 and 24 hours after treatment with 1 to 80 µM simvastatin, the LDLR gene expression levels were significantly increased by 2.4 to 2.7 times compared to untreated groups (Fig. 1B).

### Simvastatin Treatment Induced Significantly Earlier Onset and Longer Duration of Fecal HuNoV Shedding in Gn Pigs, Frequently with Higher Fecal HuNoV Titers

**Figure 2:**
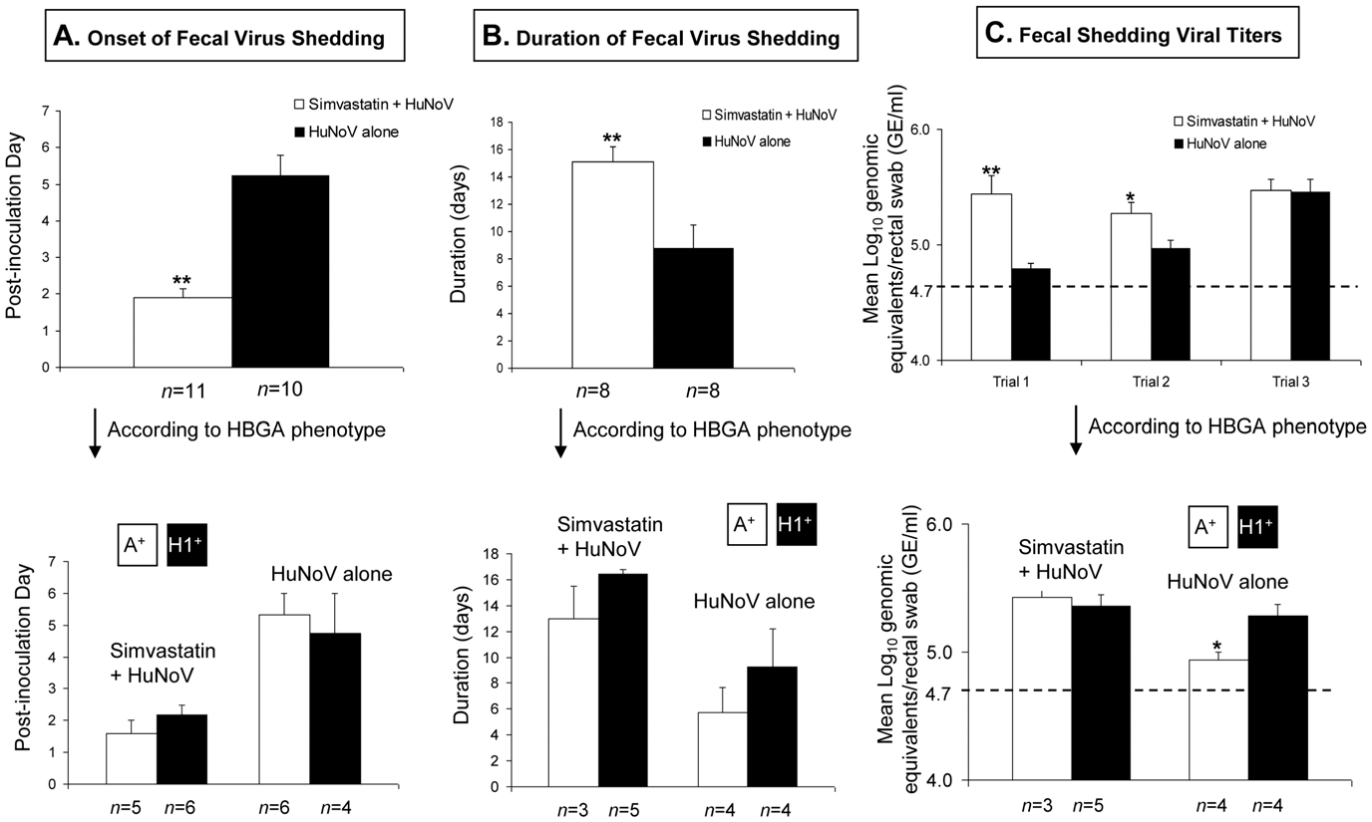
Simvastatin treatment induces significantly earlier onset and longer duration of virus shedding, frequently with higher viral titers in the feces, regardless of HBGA phenotype (A/H1) of the pigs. The mean onset and duration of fecal HuNoV shedding are summarized (**A, B**), and the daily viral titers (mean) as monitored by qRT-PCR are shown (**C**). Monitoring continued until 3 to 4 weeks after infection and terminated when PCR results were negative (<4.7 log_10_ GE/ml) for 3 consecutive days. Data from 3 independent animal trials were combined, and the PCR test was performed in duplicate or triplicate. Additional analysis was also conducted according to the HBGA A or H1 phenotype of each animal. Each bar represents the mean ± SEM. *P<0.05; **P<0.01 for Simvastatin + HuNoV vs HuNoV alone or for A+ pigs vs H+ pigs by the unpaired two-tailed Mann-Whitney test. Animal numbers (*n*) are indicated at the bottom of each graph. The dotted line indicates the detection limit (4.7 log_10_ GE/ml) of the qRT-PCR.

Significantly earlier onset of HuNoV shedding was observed in simvastatin-treated pigs, which began shedding at mean post-inoculation day (PID) 1.9±0.2, compared to mean PID 4.8±0.7 in untreated pigs (*P*<0.01) (Fig. 2A). Significantly longer duration of HuNoV shedding was also observed in simvastatin-treated pigs, which shed for a mean of 15.1±1.1 days, compared to a mean of 8.8±1.7 days in untreated pigs (*P*<0.01) (Fig. 2B). The mean daily fecal HuNoV titers were compared statistically between simvastatin-treated and untreated pigs in each trial, due to high variability in mean viral titers among the 3 independent trials. Significantly higher viral RNA titers were detected in simvastatin-treated pigs than untreated pigs in Trial 1 (5.44±0.16 log_10_ genomic equivalents (GE)/ml vs. 4.79±0.05 log_10_ GE/ml] (*P*<0.01) and Trial 2 (5.27±0.10 log_10_ GE/ml vs. 4.97±0.07 log_10_ GE/ml) (*P*<0.05) (Fig. 2C). However, no such significant difference was observed in Trial 3.

**Figure 3:**
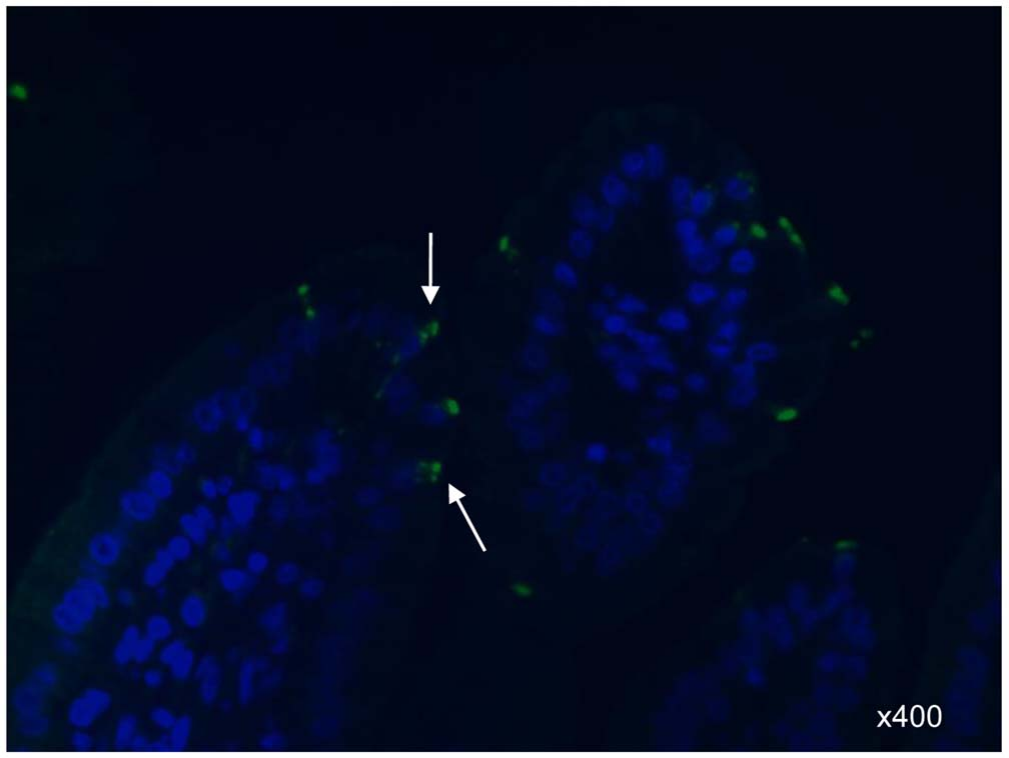
Fecal shedding coincides with IHC evidence for human NoV replication in the intestine. Jejunum of a Simvastatin + HuNoV pig at PID 3, showing viral antigens (arrows) in the cytoplasm of enterocytes. An IHC using guinea pig antiserum against the capsid protein VP1 of GII.4 HS194 HuNoV was used for detection of HuNoV antigen in formalin-fixed, paraffin-embedded tissues. Nuclei were stained with blue-fluorescent DAPI. Original magnification, x400.

**Figure 4:**
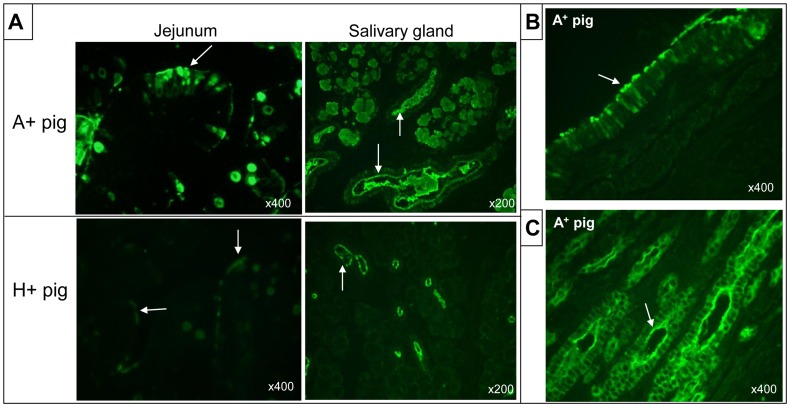
*In situ* expression of histo-blood group antigens in Gn pigs. (**A**) Jejunum and salivary gland of A^+^ or H^+^ pigs, expressing large amounts of HBGA antigens (arrows). (**B**) Bronchus of a representative A^+^ pig, expressing large amounts of A antigens on the surface or in the cytoplasm of bronchial epithelial cells. (**C**) Renal tubule of a representative A^+^ pig, expressing large amounts of A antigens on the surface or in the cytoplasm of renal tubular epithelial cells. Original magnifications, x200 or x400, are indicated at the bottom of each picture.

Immunohistochemistry (IHC) results showed HuNoV antigens in the cytoplasm or on the surface of enterocytes, but not in lamina propria cells (Fig. 3), supporting that fecal virus shedding is a result of HuNoV replication and infection in the intestine. The IHC-positive cells were in the small intestine, but not the large intestine, in which epithelial cells also expressed similar levels of HBGA as in the small intestine (Fig. 4A). However, after HuNoV inoculation of simvastatin-treated or untreated Gn pigs, no pronounced histological changes were evident in the small and large intestines of Gn pigs or as a side-effect after oral treatment with high-doses of simvastatin in controls. Under our experimental conditions no HuNoV-infected pigs showed diarrhea, whereas mild diarrhea was noticeably observed in all of the statin-treated pigs up to 5 days after statin treatment.

### Both HBGA A+ and H+ Type Pigs were Equally Susceptible to GII.4 HuNoV Infection

All Gn pigs used in this study were positive for either HBGA A or H1 (Fig. 4A–C). The HBGA antigens were distributed on the surface or in the cytoplasm of epithelial cells lining the intestine, the salivary glands, and pulmonary (bronchial) and renal tubular epithelial cells (Fig. 4A–C). Under similar IHC conditions, amounts of A antigens in A^+^ pigs were greater in the intestine and other positive tissues, as compared to those of H1 antigens in H^+^ pigs (Fig. 4A). However, no significant differences in the onset and duration of fecal virus shedding were found between the A^+^ and H^+^ pigs (Fig. 2A and B). Despite the higher expression levels of A antigens, significantly lower viral RNA titers (4.94±0.05 log_10_ GE/ml) were detected in the feces of A^+^ pigs than that (5.28±0.09 log_10_ GE/ml) in the H^+^ pigs (*P*<0.05), but no difference was observed between the simvastatin-treated, A^+^ and H^+^ pigs (Fig. 2C).

### Simvastatin Impaired TLR3-mediated Induction of IFN-α in Macrophages or Dendritic Cells, Possibly Due to Lowered Expression of TLR3 after Treatment

**Figure 5:**
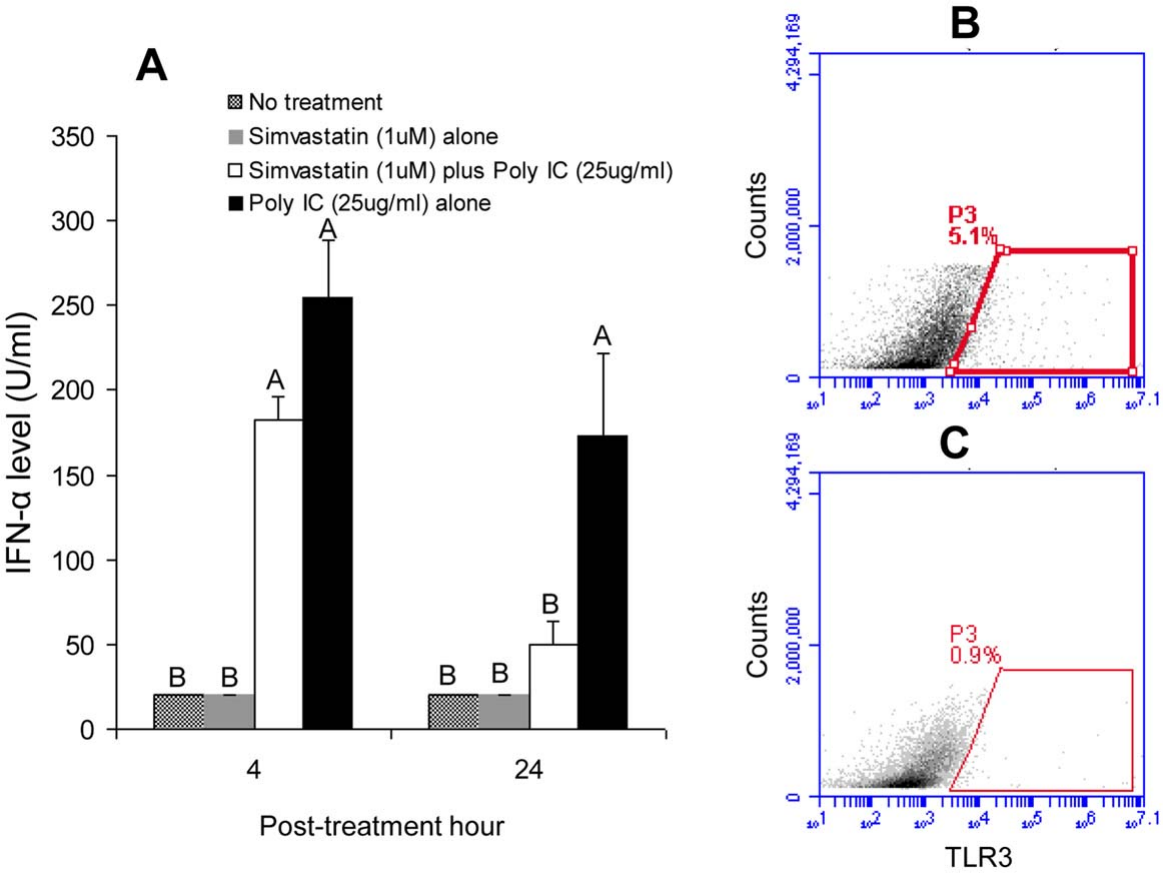
Simvastatin impairs TLR3-mediated induction of type I IFN and diminishes IFN-α levels from pulmonary macrophages (PAMs) *in vitro,* possibly due to lowered expression of TLR3 after treatment. The PAMs (2∼4×10^6^ cells/ml) were first treated with 1 µM simvastatin, and 24 hours later, were treated with either simvastatin (1 µM) or poly (I:C) (25 µg/ml), or treated with both. The cell culture supernatants were harvested to measure IFN-α levels (**A**) by biological assay at 4 and 24 hours afters poly (I:C) treatment. Each bar represents the mean ± SEM. Different letters denote significant differences among groups at each time-point (ANOVA test, *P*<0.05). (**B, C**) Flow cytometry of TLR3^+^ cells after simvastatin treatment. PBMC-derived macrophages (2∼5×10^6^ cells/ml) were first treated with 1 µM simvastatin, and 24 hours later, were treated with either simvastatin (1 µM) or poly (I:C) (25 µg/ml), or both. Cells were harvested at 24 hours afters poly (I:C) treatment and stained intracellularly for TLR3. (B) represents cells from poly (I:C) alone treatment and (C) represents cells from simvastatin + poly (I:C) treatment. A total of 5×10^4^ events were analyzed.

Our *in vivo* data showing enhanced early infectivity of HuNoV suggested potential subversion of innate immunity related to simvasatin treatment. Thus, we further investigated if simvastatin inhibits the capacity of macrophages or dendritic cells (DCs) to produce IFN-α after stimulation with poly (I:C), which triggers TLR3-mediated induction of IFN-α. In general, IFN-α was not detected in culture supernatants of macrophages or DCs treated with either simvastatin only or mock. In porcine pulmonary alveolar macrophages (PAMs), IFN-α levels (50.0±13.4 U/ml) released in simvastatin + poly (I:C)-treated PAMs were significantly lower than those (173.3±48.1 U/ml) of poly (I:C) only-treated PAMs at 24 hours after poly (I:C) treatment (*P*<0.05) (Fig. 5A). Peripheral blood mononuclear cell (PBMC)-derived macrophages responded less to treatment with 50 µg/ml of poly (I:C), as compared to PAMs treated with 25 µg/ml of poly (I:C) (Table 1), possibly due to lower ratios of harvested adherent macrophages from PBMC. Similar to the observations for PAMs, significantly lower IFN-α levels (68.9±12.9 U/ml) were observed in simvastatin-treated, enriched intestinal DCs at 12 hours after poly (I:C) treatment (*P*<0.05), as compared with those (93.3±15.3 U/ml) after poly (I:C) treatment alone (Table 1). The mean percentages (± SEM) of TLR3+ cells (3.22±0.85%, *n* = 6) from simvastatin + poly (I:C)-treated intestinal macrophages at 24 hours after poly (I:C) treatment were significantly lower (*P*<0.01), as compared to that (6.56±0.47%, *n* = 6) from poly (I:C) alone. A representative flow cytometric profile is illustrated in Fig. 5B and C.

**Table 1:** IFN-α levels (U/ml) released from intestinal and PBMC-derived macrophages or dendritic cells treated with either simvastatin (1 µM) + poly (I:C) (50 µg/ml) or poly (I:C) (50 µg/ml) alone.

Post-treatment hour	Mean IFN-α levels (± SEM)^a^ in U/ml							
	Intestinal macrophages		PBMC-derived macrophages		Intestinal DCs		PBMC-derived DCs	
	Simvastatin + poly(I:C)^b^	Poly (I:C) alone	Simvastatin + poly(I:C)	Poly (I:C) alone	Simvastatin + poly(I:C)	Poly (I:C) alone	Simvastatin + poly(I:C)	Poly (I:C) alone
12	20c,A	20^A^	57.3±7.9^A^	60.0±13.2^A^	68.9±12.9^B^	93.3±15.3d,A	102.2±16.4^A^	97.1±14.2^A^
24	20^A^	20^A^	39.5±10.1^A^	37.3±7.8^A^	40.0±0.0^A^	40.0±0.0^A^	76.2±3.8^A^	82.8±16.8^A^
36	20^A^	20^A^	43.3±9.7^A^	41.7±10.1^A^	56.7±61.4^A^	61.4±8.2^A^	55.8±12.5^ A^	60.0±8.2^A^

a Mean (U/ml) ± SEM (*n* = 6–12) performed in three separate experiments.

b Since IFN-α was not detectable (<20 U/ml) in cells treated with either simvastatin alone or mock, these two treatment groups are not shown in the table.

c Detection limit of the IFN-α assay was 20 U/ml.

d Different capital letters denote significant differences among groups within a cell type at each time-point (ANOVA test, *P*<0.05).

### Neither the Interferon Regulatory Factor 3 (IRF3) Nor Nuclear Factor Kappa B (NFκB) Signaling Pathway Appeared to be Involved in Decreased IFN-α Responses Induced by Simvastatin

**Figure 6:**
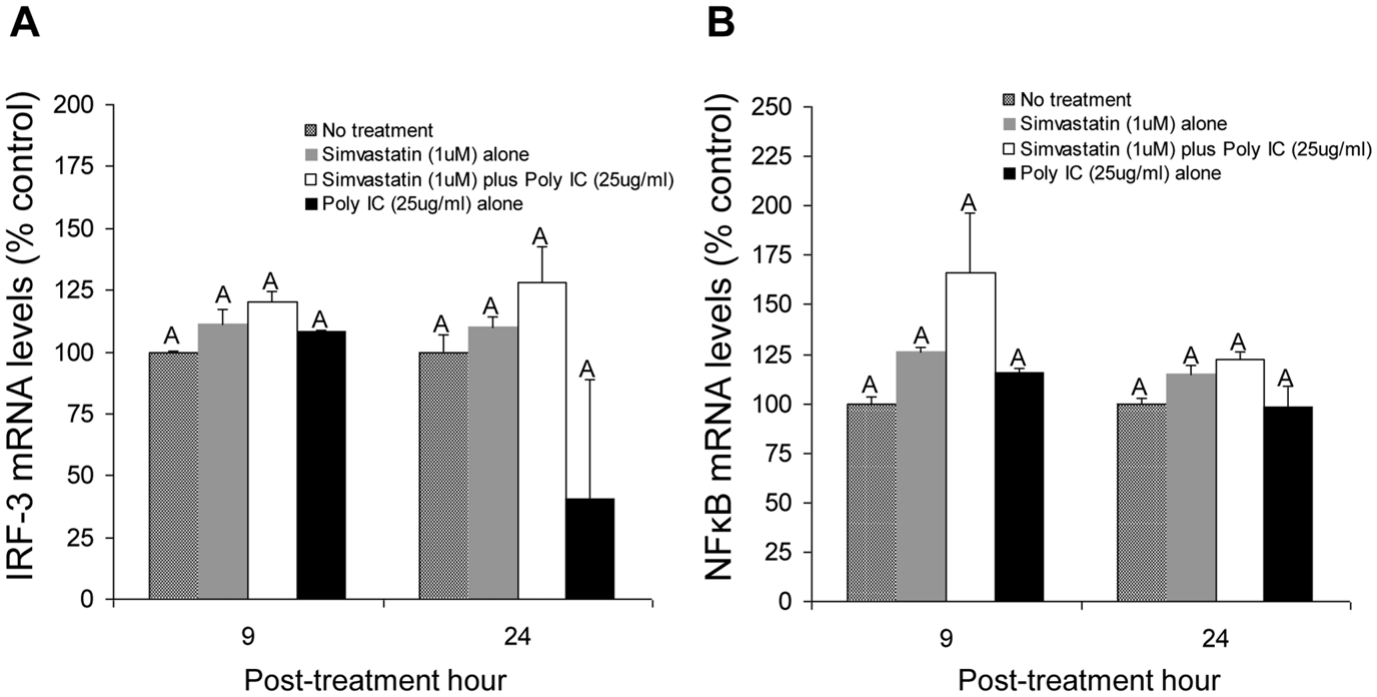
Neither the IRF3 nor NFκB singaling pathway appears to be involved in decreased IFN-α responses induced by simvastatin. mRNA expression levels of IRF3 (**A**) and NFκB (**B**) in simvastatin-treated macrophages at 9 and 24 hours after poly (I:C) stimulation. The mRNA expression levels were measured by qRT-PCR and normalized to the expression levels of β-actin. Each bar represents the mean ± SEM. Data from 2 independent experiments were combined, and each test was performed in triplicate.

Gene expression levels of IRF3 and NFκB, mainly involved in poly (I:C)-induced, TLR3-mediated IFN production, were analyzed to investigate possible mechanisms underlying the subversion of innate immunity induced by simvastatin in PAMs and intestinal DCs. Although simvastatin alone did not induce IFN-α production in PAMs (Fig. 5A), increased expression of IRF3 and NFκB genes was found in simvastatin-treated PAMs (Fig. 6A and B). Co-treatment with simvastatin and poly (I:C) synergistically resulted in increased gene expression of IRF3 and NFκB. At 24 hours after poly (I:C) treatment, however, gene expression levels of IRF3 and NFκB were reduced in poly (I:C) only-treated PAMs, but not in simvastatin + poly (I:C)-treated PAMs, as compared to no treatments.

### HuNoV Fecal Shedding in Infected Pigs was Reduced or Curtailed during Oral Treatment with Natural Human IFN-α

**Figure 7:**
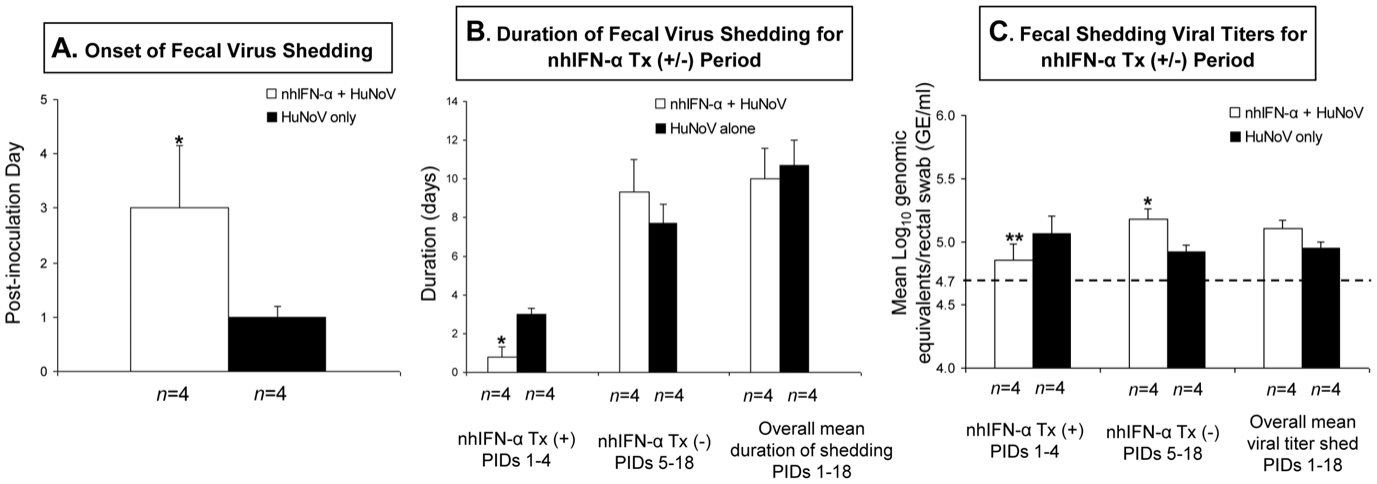
HuNoV fecal shedding in infected pigs can be reduced or curtailed during oral treatment with natural human IFN-α. The mean onset and duration of fecal HuNoV shedding are summarized (**A, B**), and the daily viral titers (mean) as monitored by qRT-PCR are shown (**C**). Five or six day-old piglets were treated orally with 300 IU of nhIFN-α once a day from PID -1 to PID 4. On day 2 after nhIFN-α treatment, they were inoculated orally with GII.4 HS194 HuNoV, and subsequently treated with nhIFN-α (300 IU) for 5 more days. After nhIFN-α treatment or HuNoV inoculation, clinical signs and fecal virus shedding were monitored daily until shedding terminated. Data from 2 independent animal trials were combined, and the PCR test was performed in duplicate or triplicate. Duration of virus shedding in nhIFN-α-treated and untreated pigs were analyzed based on nhIFN-α treatment period, i.e. during treatment at PIDs 1–4; post-treatment at PIDs 5–18; and overall at PIDs 1–18. Each bar represents the mean ± SEM. *P<0.05; **P<0.01 for nhIFN-α + HuNoV vs HuNoV alone by the unpaired two-tailed Mann-Whitney test. Animal numbers (*n*) are indicated at the bottom of each graph. The dotted line indicates the detection limit (4.7 log_10_ GE/ml) of the qRT-PCR.

Swine IFN-α levels were decreased in the poly (I:C) and simvastatin-treated PAMs or DCs, and HuNoV infection was enhanced *in vivo*. Therefore, we investigated whether fecal HuNoV shedding, i.e. HuNoV replication in the gut of infected Gn pigs, was altered by treatment with IFN-α. Oral treatment of Gn pigs with natural human IFN-α (nhIFN-α) [300 international unit (IU)/kg/day] reduced or curtailed virus shedding in treated animals during the treatment period (PID 1 to 4), compared to untreated animals (Fig. 7A–C). The treatment significantly delayed the onset of virus shedding by 1.7 day in treated pigs, which began shedding at mean PID 3.0±0.8, compared to mean PID 1.3±0.2 in untreated pigs (*P*<0.05) (Fig. 7A). During the nhIFN-α treatment period (PID 1 to 4), a significantly shorter duration of HuNoV shedding was observed in the nhIFN-α-treated pigs, which shed for a mean of 0.8±0.5 days, compared to a mean of 2.0±0.3 days in untreated pigs (*P*<0.05) (Fig. 7B). During the treatment period, a significantly lower qRT-PCR-positive rate of the fecal samples tested (*P*<0.01) was also observed in the nhIFN-α-treated pigs (3/16; 18.8%) than in the untreated pigs (12/16; 75%), with significantly lower viral RNA titers in the feces (4.88±0.11 log_10_ GE/ml in the treated pigs vs. 5.06±0.14 log_10_ GE/ml in the untreated pigs) (*P*<0.01) (Fig. 7C). However, at PID 5 to 18 after nhIFN-α-treatment was discontinued, significantly increased viral shedding titers were noted in the nhIFN-α-treated pigs (5.18±0.08 log_10_ GE/ml), compared to the untreated pigs (4.92±0.05 log_10_ GE/ml) (*P*<0.05) (Fig. 7C). At PIDs 5–18, there were no significant differences in the duration of fecal virus shedding and the qRT-PCR-positive rate of the fecal samples tested between the nhIFN-α-treated pigs and untreated pigs (Fig. 7B). Further repeated studies in additional pigs are needed to investigate how termination of nhIFN treatment results in higher viral shedding titers post IFN-α treatment. No negative control pigs shed detectable viral RNA in the feces throughout the experiment.

## Discussion

We demonstrated that use of simvastatin enhances HuNoV infectivity in the Gn pig model. Thus, its use may also support growth of HuNoV in cell culture. We also verified that the increased infectivity of HuNoV may be associated with the inhibitory effect of statins on innate immunity (IFN-α). This observation might explain the exacerbated HuNoV disease and the related higher mortality described in statin-treated humans [14]. Because of the immunosuppressive effects, use of statins have been proposed for immunomodulatory therapy against severe influenza A virus infections in which large amounts of innate (IFN-α) cytokines are involved [24]. In addition, we showed that oral treatment with nhIFN-α can curtail early HuNoV fecal shedding in the Gn pig model. Because no HuNoV vaccines are available, use of effective antivirals such as IFN-α should be tested to control multiple genogroups and genotypes of HuNoVs, including the GII.4 variants that have emerged each year [25]. Our findings that HuNoV infectivity in Gn pigs can be enhanced by simvastatin treatment or reduced by oral treatment with nhIFN-α, suggest that Gn pigs are a useful model to test efficacy of antivirals against HuNoV.

As a surrogate model for HuNoVs, murine NoV (MNV) infection of mice was useful for investigating the roles of specific immunologic factors such as type I or II IFNs in host defense [15]. However, the different pathogenesis of MNV infection with its systemic spread raises concerns about extrapolation of these findings to the HuNoV restricted gastrointestinal infection. A chimpanzee model was recently established to evaluate the efficacy of VLP-derived vaccines against infection with GI or GII HuNoVs [26]. Chimpanzees developed serum antibody responses after intravenous injection of GI.1/Norwalk virus or intramuscular injection with Norwalk VLPs and were protected from GI.1/Norwalk virus challenge (but not GII HuNoV infection). The chimpanzee model, however, is compromised by the lack of availability of chimpanzees, and the finding that oral infection of chimpanzees with HuNoVs failed to induce gastroenteric disease comparable to human cases [26], as well as by the intravenous route required for viral challenge. In our study, although HuNoV infection of Gn pigs induced mild enteric disease, Gn pigs were susceptible to oral infection by the GII.4 HS194 strain, which reaffirms the results of our previous studies using a closely related GII.4 HuNoV (HS66 strain) [16], [17] and the emerging GII.12 HuNoV (HS206 strain) [11]. Fecal HuNoV shedding patterns in Gn pigs, with peak viral titers during an early stage of infection are also typical for other acute enteric viral infections in pigs, such as rotavirus and porcine epidemic diarrhea virus [27], [28]. However, how HuNoV shedding in infected Gn pigs is maintained for 2 or 3 weeks after viral inoculation is unclear and requires further investigation.

Cholesterol biosynthesis and metabolism are mainly mediated by hepatic enzymes, such as HMG-CoA reductase [29]. Statins act as competitive inhibitors of HMG-CoA reductase and reduce production of cholesterol in the liver. As in humans, our study showed that statins lower serum cholesterol levels in Gn pigs, possibly due to similar cholesterol pathways between swine and humans as reported previously [30]. When hepatic cholesterol stores are depleted, the liver increases the expression of LDLR which leads to uptake of LDL from plasma to compensate for the lower cellular cholesterol levels. Several RNA viruses manipulate cholesterol pathways in diverse ways for more efficient viral infection and replication as exemplified for NoVs in comparion to hepatitis C virus (HCV) and coronavirus [20], [31], [32], [33]. For example, low cellular cholesterol levels (or high cellular LDLR expression) following statin treatment contributed to increased GI.1/Norwalk virus replication, as verified in an *in vitro* replicon system [20]. Our study also showed that increased LDLR expression levels in IPEC-J2 cells (a porcine jejunal cell line) treated with simvastatin might similarly contribute to enhanced HuNoV replication in the gastrointestinal tract. Our other ongoing *in vitro* studies also found that HuNoV RNA titers in supernatants or lysate samples of IPEC-J2 cells treated with simvastatin were slightly increased in trials using GII.12 HS206 strain compared to those of controls (without simvastatin), but did not differ significantly in cell cultures using the GII.4 HS194 strain. The latter was previously reported [34]. The data indicate a positive but inconsistent effect of simvastatin on HuNoV replication *in vitro*, possibly depending on the HuNoV strains or different environmental conditions for HuNoV replication *in vitro* versus *in vivo*. A paper describing more detailed and comprehensive *in vitro* cell culture findings is in preparation by Takanashi et al. (unpublished data, 2012). In addition to the cholesterol lowering effects, the inhibitory effects of statins on innate immunity also might influence the immunological and cellular microenvironment for more efficient HuNoV replication. In our study, simvastatin impaired TLR3-mediated innate immunity and inhibited production of IFN-α induced by poly (I:C) in PAMs or intestinal DCs. These observations are similar to the results of an *in vitro* study using HEK-293 cells, showing the inhibitory effect of simvastatin on TLR4-mediated immune responses, such as tumor necrosis factor (TNF)-α and interleukin-6 [23]. Nevertheless, it is notable that observations for Gn pigs and humans infected with HuNoVs [14] are contrary to the effect of statins that reduced replication of HCV in replicon-harboring cells [31] and a positive correlation between cellular cholesterol levels and entry of coronaviruses [32] and of GV/MNV into host cells [33]. Further confirmatory data are needed to define the role of the cholesterol pathway in the pathogenesis of HuNoV.

Although simvastatin treatment inhibited IFN-α production, we found that gene expression of IRF3 and NFκB in simvastatin-treated PAMs was increased rather than being decreased. Because activation of NFκB kinase is a shared property among TLRs, including TLR3 [35], simvastatin or its cellular byproducts could trigger other TLRs that stimulate NFκB gene expression. Notably, at 24 hours after poly (I:C) treatment, gene expression levels of IRF3 and NFκB in poly (I:C) only-treated cells were reduced remarkably, as compared with other treatments or those at the earlier time-point. This observation could be explained by a cellular negative feedback effect to mediate production of IFN-α. It is also notable that a similar result did not occur in simvastatin + poly (I:C)-treated cells, possibly due to reduced IFN-α levels after simvastatin treatment. At least four families of transcription factors are activated by dsRNA and relate to TLR3: NFκB, IRF-3, c-Jun, and activating transcription factor 2 [35]. Besides lowered TLR3 expression by macrophages after simvastatin treatment, which was thought to be mainly responsible for reduction of IFN-α production in statin + poly (I:C)-treated cells, other TLR3- or IFN-mediated signaling pathways might be involved in the impaired innate immunity by simvastatin.

Type I IFNs are essential for early viral clearance and development of adaptive immune responses. As a crucial mediator of the innate antiviral immune responses, IFN-α has been an effective antiviral treatment for viral infections, such as HCV and influenza [36], [37]. The signal transducer and activator of transcription-1 (STAT-1)-dependent IFN stimulation was essential for controlling murine NoV (MNV) infection. Although MNV did not cause disease in immunocompetent mice, oral MNV infection caused fatal systemic disease in mice lacking either type I and type II interferon receptors or STAT-1, which is critical for IFN signaling [15], [38]. Several studies have suggested that repeated oral treatment with nhIFN-α may be effective in treating acute viral gastroenteritis related to coronavirus and rotavirus in domestic pigs [39], [40]. Similarly, our study showed that oral administration of nhIFN-α inhibits infection by or replication of HuNoV, as fecal HuNoV shedding is curtailed in the Gn pig model. The nhIFN-α is formulated to be stable at low pH of the stomach. Degradation of nhIFN-α by a variety of intestinal enzymes appears to be slow enough to allow nhIFN-α to reach some IFN-α receptors of cells in mucosal lymphoid tissues of the oral cavity and intestine [41]. The therapeutic effectiveness of nhIFN-α might be related to its immunostimulatory effects. Orally delivered nhIFN-α promoted systemic innate immunity by increasing expression levels of innate immunity-related genes, such as IFN-stimulated genes (ISGs) and TNF-α, and phagocytic capacity of phagocytes [42], [43]. Further studies with larger numbers of animals are needed to determine the most effective dose and regimen of nhIFN-α to prevent or treat HuNoV infections, and to elucidate the immunological and molecular mechanisms related to the antiviral effects of IFN-α. Based on the effectiveness of a combination of nhIFN-α pre- and post-treatment, the nhIFN-α treatments need to be tested therapeutically in future studies using the Gn pig model or in clinical trials. The mechanisms by which fecal virus shedding recurred and the increased viral RNA titers in nhIFN-treated Gn pigs compared to untreated pigs after nhIFN treatment was discontinued need to be investigated. However, we hypothesize that during the period of nhIFN treatment, IFN signaling pathways might be regulated by a negative feedback in some IFN producing cells in the intestine. Thus, on the termination of treatment such a distinct condition of the IFN system might hinder induction or production of IFN-α in most IFN containing cells or its antiviral activity against HuNoV.

In conclusion, simvastatin treatment increased HuNoV infectivity in the Gn pig model, possibly due to its inhibitory effect on innate immunity as well as its cholesterol lowering effect as reported previously [20]. These findings could partially explain the exacerbated HuNoV disease in statin-treated humans [14]. Testing of nhIFN-α as an antiviral for HuNoV using the Gn pig model also revealed that IFN-α has potential as a HuNoV antiviral therapy. Development of HuNoV antivirals is important because HuNoVs cause large-scale epidemics with significant mortality in immunocompromised, elderly and young patients. Thus, the Gn pig model for HuNoV will allow testing of new treatment modalities for HuNoV infection and new knowledge on the antiviral mechanisms of innate and adaptive immunity.

## Materials and Methods

### Cell and Virus

The IPEC-J2 cells were kindly provided by Dr. Bruce D. Schultz (Kansas State University) [44]. Cells were maintained in Dulbecco’s Modified Eagle’s Medium/Nutrient Ham’s Mixture F-12 (Invitrogen, Carlsbad, CA) with 5% fetal bovine serum (FBS; HyClone Laboratories, Inc., Logan, UT), 1% insulin-transferrin-sodium selenite (Roche, Mannheim, Germany), and epidermal growth factor (5 ng/ml) (Invitrogen). The GII.4/HS194/2009/US (HS194) strain (GenBank accession number: GU325839) used as viral inoculum in this study was isolated from stool samples of a young child with watery diarrhea [11]. Stool samples were screened for other enteric viruses, including GI HuNoV, rotavirus groups A, B and C, sapovirus, astrovirus, and adenovirus by reverse transcription (RT)-PCR or PCR, respectively, as described previously [11].

### Pigs and A/H Phenotyping

Near-term pigs were derived by hysterectomy and maintained in sterile isolator units [16]. By IHC using monoclonal antibodies to human A (Immucor, Norcross, CA) and H1 (Covance Research Products, Inc., Dedham, MA), we determined the A/H phenotype on fresh bucal cells or formalin-fixed, paraffin-embedded intestinal and salivary glandular tissues of Gn pigs. The Institutional Animal Care and Use Committee (IACUC) of the Ohio State University approved all protocols related to the animal experiments in this study. All animals used in this study were also handled in accordance with the guidelines of the IACUC of the Ohio State University.

### Experimental Pig Infection and Treatment with Simvastatin

Piglets were randomly assigned to one of four groups: Simvastatin + HuNoV (*n = *11), HuNoV alone (*n = *10), Mock (*n = *4), and Simvastatin alone (*n = *4). To lower serum cholesterol levels prior to virus infection, five or seven day-old piglets were first treated orally with 8 mg/day/pig (approximately 1 kg of body weight) of simvastatin (Zocor; Merck and Co, Inc., Whitehouse station, NJ). On day 6 after treatment, they were infected orally with 2.4×10^9^ or 3×10^10^ GE of the GII.4 HuNoV HS194, and then subsequently treated with the half doses of pre-treatment for 5 more days. After simvastatin treatment or HuNoV inoculation, we monitored clinical signs daily. At an acute (PID 3 to 5) stage of HuNoV infection, 1 to 4 pigs per group were euthanized for histopahological examination. When virus fecal shedding terminated as determined by qRT-PCR, i.e. at a later (PID 20 to 29) stage of infection, 2 to 8 pigs per group were euthanized.

### Treatment of Pigs with nhIFN-α

The nhIFN-α was kindly provided by Dr. Joseph Cummins (Amarillo Biosciences, Inc., Amarillo, TX). Piglets were randomly assigned to one of three groups and constituted 2 independent trials: nhIFN-α-treated, HuNoV-infected (*n = *4), nhIFN-α-untreated, HuNoV-infected (*n = *4), and negative control (*n = *4). Five or six day-old piglets (approximately 1 kg of body weight) were treated orally with 300 IU of nhIFN-α once a day from PID -1 to PID 4. On day 2 after nhIFN-α treatment, they were infected orally with 1.3×10^10^ GE of the GII.4 HS194 HuNoV, and subsequently treated with nhIFN-α (300 IU) for 5 more days. After nhIFN-α treatment or HuNoV inoculation, clinical signs and fecal virus shedding were monitored daily until shedding terminated.

### Cholesterol Assay

Total serum cholesterol levels were assessed in simvastatin treatment trials by using an Amplex Red cholesterol assay kit (Invitrogen), as described previously [20]. Total cholesterol was extracted in chloroform–methanol–double-distilled water [(4∶2∶1) (vol/vol/vol)]. The chloroform phase was separated, mixed with a 1∶100 volume of polyoxyethylene 9-lauryl ether (Sigma-Aldrich, St. Louis, MO), dried, and resuspended in the assay reaction buffer in the kit. Each treatment was duplicated in additional six-well plates, and cell lysates were prepared for the measurement of protein contents by using a BCA protein assay kit (Bio-Rad, Hercules, CA). The concentrations of total cholesterol were normalized with the protein contents.

### Analysis of HuNoV Fecal Shedding Titer

Rectal swabs were collected daily from each animal throughout the experiment. The GII HuNoV fecal shedding titers were determined by the TaqMan real-time RT-PCR (COG2F/2R primer set and RING2 probe), as described previously [11], [45]. The detection limit of this PCR assay was 10 GE per reaction determined based on the standard curve generated using serially diluted plasmid DNA carrying HS194-specific COG2F/2R amplicons. The limit of viral RNA detection in the qRT-PCR assay was 4.7 log_10_ GE/ml.

### Expression and Purification of the Virus-like Particles (VLPs) of Hu/NoV/GII.4/HS194/2009/US Strain and Production of Hyperimmune Serum

The recombinant baculovirus carrying the capsid protein (VP1) gene (ORF2) of HS194 strain (GenBank accession number: GU325839) was generated by using the BaculoDirect™ Baculovirus Expression System (Invitrogen) according to the manufacturer’s instructions. Briefly, a Gateway entry clone containing the ORF2 of HS194 (pENTR™/SD/D-TOPO-HS194) was generated and used with the BaculoDirect™ Linear DNA to perform a LR recombination reaction to generate recombinant baculovirus DNA carrying the ORF2 of HS194. The insect Sf9 cells were transfected by the recombination reaction products and the cells containing the recombinant baculovirus DNA were positively selected by ganciclovir. The expression of HS194 capsid proteins in the Sf9 cells and culture supernatants were examined by immunoblot using the guinea pig antiserum against Hu/NoV/GII.4/HS66/2001/US strain [46]. The recombinant baculoviruses (rBac-HS194) were propagated to prepare virus stocks with high titers for routine VLP expression. The production and purification of HS194 VLPs were performed as described previously [46]. The protein concentration was quantified using the Bradford method and the VLPs were negatively stained with 3% phosphotungstic acid (pH 7.0) and examined by transmission electron microscopy as described previously [11]. Hyperimmune serum against HuNoV GII.4 HS194 VLPs was generated using guinea pigs according to an approved IACUC protocol, as previously described [16].

### Histological Analysis and Immunohistochemistry for HuNoV Antigen Detection

Small (duodenum, proximal, middle and distal jejunum, and ileum) and large (cecum and colon) intestinal tissues and other major organs (lung, liver, heart, kidney, spleen, and lymph node) were examined grossly and histologically and tested by IHC for NoV antigen detection. Tissues from age-matched mock controls were tested for histological comparisons and as a negative control for IHC. The IHC was performed on formalin-fixed, paraffin-embedded tissues or fresh frozen tissues using the guinea pig hyperimmune antisera to VLPs of the GII.4 HuNoV HS194, as described previously [47].

### Treatment of Alveolar Macrophages with Simvastatin or Poly (I:C)

The PAMs (2∼4×10^6^ cells/ml) were collected from the lungs of 5 ten-day-old, uninfected Gn pigs using aseptic techniques, as previously described [48], [49]. Cells (4∼8×10^5^ cells/well) were seeded onto 12-well cell plates, and the wells were randomly assigned to 4 treatment groups: No treatment, Simvastatin alone, Simvastatin + poly (I:C), and Poly (I:C) alone. PAMs were first treated with 1 µM simvastatin (Sigma), and 24 hours later, treated with either 1 µM simvastatin or poly (I:C) (25 µg/ml) (Sigma), or treated with both. The cell culture supernatants were harvested at 4 and 24 hours after poly (I:C) treatment to measure IFN-α levels released from PAMs.

### Treatment of Intestinal and PBMC-derived Macrophages and Dendritic Cells with Simvastatin or Poly (I:C)

Intestinal mononuclear cells or PBMC were isolated from ileum or blood of Gn pigs, as previously described [27]. Gut or PBMC monocyte-derived macrophage or DC-enriched cell preparations were obtained by plating gut mononuclear cells or PBMC at 2∼4×10^6^ cells/ml in RPMI-1640 supplemented with 8% FBS, 1% gentamicin, 0.1% ampicilin, 20 mM HEPES, 2 mM L-glutamine, and 1 mM sodium pyruvate for 2–3 days before harvesting adherent cells. Non-adherent cells were used for DC-enriched cells. Macrophage- or DC-enriched cells (4∼8×10^5^ cells/well) were seeded onto 12-well cell plates, and the wells were randomly assigned to 4 treatment groups: No treatment, Simvastatin alone, Simvastatin + poly (I:C), and Poly (I:C) alone. Cells were treated with simvastatin in the same manner as for PAMs.

### IFN-α Detection by Biological Assay

Mardin-Darby bovine kidney (MDBK) cells were grown in MEM with 5% FBS and 1% antibiotic-antimycotic. The cell supernatant samples, serially diluted 1∶2 in MEM, were added to the confluent cell monolayers seeded in 96-well plates, as previously described [50]. At 24 hrs after incubation at 37°C, media was removed and 100 µl of vesicular stomatitis virus (3×10^5^ plaque forming unit/ml) was added. At 48 hrs after incubation, 10 µl of alamar blue was added to each well and incubated for 3 hrs at 37°C. Fluorescence was measured at 530–560 nm. Antiviral IFN-α levels (U/ml) were expressed as the reciprocal of the sample dilution which resulted in a 50% reduction in cytopathic effects.

### Measurement of mRNA Levels of LDLR, NFκB, and IRF3

Expression levels of porcine LDLR, NF*κ*B, and IRF3 mRNA were measured in IPEC-J2 cells or PAMs by Taqman real-time PCR, as described previously [51], [52], [53], with slight modifications. The mRNA expressions were normalized to the expression levels of porcine β-actin [54]. Primers and probes of LDLR and β-actin and probes of NFκB and IRF3 were designed by Geneious primer design software, as follows (5′–3′): LDLR F, CGCCCTCCAAAACGGTGGCT; LDLR R, ACTTCGGCGAGCGTGGGTTG; and LDLR probe, FAM-ACCTGTGTCTGCCAGCTCCACA-3IABkFQ. β-actin F, CCCACGCCATCCTGCGTCTG; β-actin R, GTAGCCCCGCTCCGTCAGGA; and probe, FAM-GGCCGGGACCTGACCGACTA-3IABkFQ. NFκB probe, FAM-ACCAGGCTGGCAGCTCTCCTCAAAGCAGCA-3IABkFQ. IRF3 probe, FAM-CCGGTCTGCCCTGAACCGGAA-3IABkFQ.

### Statistical Analysis

All values are expressed as the means ± standard error of the means (SEM). Cholesterol level data among the treatment groups were analyzed by the Kruskal-Wallis test (nonparametric) using the Statistical Analysis Systems. All gene expression and IFN-α level data and numbers of TLR3+ cells were analyzed by one-way analysis of variance (ANOVA). Virus titers that were undetectable (<4.7 log_10_ GE/ml) during the shedding period were assigned as a value of 4.7 log_10_ GE/ml for statistical analysis. The mean onset and duration of virus shedding and viral titers between Simvastatin + HuNoV and HuNoV alone groups, between A^+^ and H^+^ pigs, and between nhIFN-α-treated and untreated pigs, were compared by unpaired two-tailed Mann-Whitney tests. Specifically, duration of virus shedding and viral titers in nhIFN-α-treated and untreated pigs were analyzed based on nhIFN-α treatment period, i.e. during treatment at PIDs 1–4; post-treatment at PIDs 5–18; and overall at PIDs 1–18. Fisher’s exact test was used to compare the PCR-positive rates of the fecal samples tested at PIDs 1–4 or PIDs 5–18 between the nhIFN-α-treated and untreated pigs. A value of *P*<0.05 was considered statistically significant.
